# Large-Scale Multi-Omic Approaches and Atlas-Based Profiling for Cellular, Molecular, and Functional Specificity, Heterogeneity, and Diversity in the Endocannabinoid System

**DOI:** 10.3390/cells12050814

**Published:** 2023-03-06

**Authors:** Jun Aoki, Masako Isokawa

**Affiliations:** 1 Graduate School of Frontier Biosciences, Osaka University, Osaka 565-0871, Japan; 2 Laboratory for Cell Signaling Dynamics, Center for Biosystems Dynamics Research, RIKEN, Kobe 650-0047, Japan; 3 Forefront Research Center, Graduate School of Science, Osaka University, Osaka 560-0043, Japan

The endocannabinoid system (ECS) is a widely-recognized lipid messenger system involved in many aspects of our our lives in health and diseases. The system consists of cannabinoid receptors, their endogenous ligands, and the enzymes that mediate their synthesis and metabolic processes. Research progress was recently reviewed regarding (i) the pharmacology of the ECS, (ii) the roles of ECSs in development and synaptic function, (iii) cannabinoid signaling in pathological conditions, and (iv) cell-type-specific and localization-dependent operations of cannabinoid receptors [1,2]. Of particular interest may be the conceptual framework where biomedical consequences influenced by ECS are not necessarily monotheistic; instead, there exists cellular, molecular, and functional specificity, heterogeneity, and diversity in (endo)cannabinoid action. 

This Special Issue aims to discuss the multiplexity of endocannabinoid signaling mechanisms and functions considering large-scale multi-omic approaches and the way data are visualized for presentation. It is well accepted that lipidomic [3], transcriptomic [4], proteomic [5], or any combination of these data mining strategies and atlas-based data profiling methods [6,7] helped identify the involvement of critical molecules throughout our lives. Methodological orientation [8,9,10] provided typical standards for non-parametric dimensionality reduction, data visualization, and cluster analysis that ranged from traditional principal component analysis (PCA) to the currently popular uniform manifold approximation and projection (UMAP) method. Some researchers may feel that these approaches are still young and relatively underexplored in endocannabinoid research; however, fruitful applications have already emerged in the investigation of ligands, receptors, and related enzymes.

The first example concerns the role of ECSs in the transition of retinal Müller glia (MG) into proliferating progenitor-like cells in health and diseases [11,12]. Using single-cell RNA sequencing (scRNA-seq) libraries, the patterns and levels of eCB-related gene expressions across different retinal cells were presented with two-dimensional profiling methods, such as UMAP and violin plots. UMAP-ordered cells formed distinct clusters of neuronal cells, resting MG, and activated MG. The expression levels in Cnr1, MGLλ, DAGLα, DAGLβ, and FAAH were demonstrated using heatmaps, and the cell-type specificity was shown by violin plots. Furthermore, the involvement of fatty-acid-binding proteins and fatty acid synthase was shown in the formation of Müller-glia-derived progenitor cells (MGPCs) after NMDA-induced damage. UMAP analysis illustrated aggregates of damages and the formation of distinct MGPC clusters according to the expression of GLUL, RLBP1, and SLC1A3, or NESTIN, CDK1, and TOP2A. Heatmaps and violin plots showed patterns and levels of FABP5, FABP7, and PMP2. These reports strived for a fuller understanding of the contribution of ECSs and fatty acid signaling in the reactivity and dedifferentiation of Müller glia, as well as the proliferation of microglia and MGPCs.

The second example is about the involvement of cannabinoid receptor type 1 (CB1R/Cnr1) and type 2 (CB2R/Cnr2) in nonalcoholic fatty liver disease (NAFLD) [13] and melanoma [14], respectively. scRNA-seq and UMAP highlighted six clusters of transcriptomic data in main liver cells, and violin plots depicted the hepatocyte-zone-specific gene expression. In melanoma, UMAP dimensionality reduction processed 6381 B-cells and mapped the top 10 upregulated genes (highest-fold change). Although the deletion of Cnr1 (CB1R KO mice) was expected to prevent the development of NAFLD, scRNA-seq and UMAP analysis did not support the hypothesis, showing that Cnr1−/− mice failed to protect the liver from fibrosis. In melanoma, UMAP analysis portrayed a positive correlation between the upregulation of intra-tumoral CB2R gene expression and improved overall survival, as well as a reduction in the metabolic activity of tumor-infiltrating B cells in Cnr2−/− mice.

Anandamide synthesis was targeted by global transcriptomic analysis in the partial hepatectomy (PHX) model [15]. The result illuminated the up-regulation of cell-cycle proteins, such as cyclin-dependent kinase 1 (Cdk1), cyclin B2, and their transcriptional regulator forkhead box protein M1 (FoxM1), and provided molecular and genetic support to the pathophysiological observation that PHX increased biosynthesis of anandamide in the liver via conjugation of arachidonic acid and ethanolamine by fatty acid amide hydrolase.

Last but not least, it may be worth mentioning that in pharmaceutical sciences, dimensionality reduction and cluster analyses, such as t-distributed stochastic neighbor embedding (t-SNE) and UMAP, have been frequently used on large-scale samples of cannabis sativa chemotypes for the purpose of modeling cannabinoids [16]. Here, a dataset of 17,600 commercial cultivars was screened for unknown gene regulation and pharmacokinetics of dozens of cannabinoids. The concentration of tetrahydrocannabinol (THC) scattered against the concentration of cannabidiol (CBD) was plotted to segregate high- and low-CBD and -THC cultivars. These approaches not only helped reveal complex interactions in cannabinoid biosynthesis but also advanced the phenotypical classification of cannabis cultivars.

We introduced scRNA-seq, t-SNE, UMAP, heatmaps, and violin plots as examples among the popular methodological tools for data generation and analysis. scRNA-seq provides a way of comprehensively defining gene expression and identifying molecular trajectories by connecting transcriptomes. However, the reconstruction of molecular lineages from gene-expression cascades to cell-type-specific markers and regulators is still a major challenge. t-SNE, UMAP, heatmaps, dendrograms, and violin plots are the techniques in data science that reduce the dimensionality of raw data and visualize the outcomes in a pictorial format. They are routinely applied in a broad range of fields, including life sciences, where datasets of increasing sizes are handled. While these techniques have been used liberally in combination with transcriptomics and proteomics, less usage has been acknowledged in the detection of molecules that cannot be labeled with antibodies and/or genetic manipulation. Further applications are encouraged with all-inclusive measurement technologies such as imaging mass spectrometry in cells and organisms, as it can directly detect and visualize the identified and unidentified lipids and metabolites that often play key roles in the eCB system.

We hope you will find the collection of papers in this Special Issue interesting and helpful for expanding methodological choices in the event of mining, summarizing, and presenting new ideas and perspectives for future eCB research.
